# The effect of Myo‐inositol on improving sperm quality and IVF outcomes: A systematic review and meta‐analysis

**DOI:** 10.1002/fsn3.4427

**Published:** 2024-09-12

**Authors:** Marjan Ghaemi, Niloofar Seighali, Arman Shafiee, Maryam Beiky, Omid Kohandel Gargari, Alireza Azarboo, Vida Shafti, Kyana Jafarabady, Nasim Eshraghi, Mohammad Haddadi, Razieh Akbari, Zahra Panahi, Sedigheh Hantoushzadeh

**Affiliations:** ^1^ Vali‐E‐Asr Reproductive Health Research Center, Family Health Research Institute Tehran University of Medical Sciences Tehran Iran; ^2^ Student Research Committee, School of Medicine Alborz University of Medical Sciences Karaj Iran; ^3^ Department of Psychiatry and Mental Health Alborz University of Medical Sciences Karaj Iran

**Keywords:** inositol, meta‐analysis, Myo‐inositol, oligo‐astheno‐teratozoospermia, semen analysis, sperm parameters, systematic review

## Abstract

Myo‐inositol may be efficient to improve sperm parameters to increase the chance of fertility. Although, the data are controversial. This study aimed to assess the impact of Myo‐inositol supplements on semen quality and in vitro fertilization (IVF) outcomes. In this systematic review and meta‐analysis, a comprehensive search was conducted in PubMed, Web of Science, and Embase. The objective was to identify relevant human studies that investigated the effects of Myo‐inositol treatment on various sperm factors, such as sperm motility, sperm concentration, sperm morphology, viable spermatozoa, spermatozoa with DNA fragmentation, and pregnancy rate. Additionally, the testosterone levels of patients with Oligo‐astheno‐teratozoospermia (OAT) after Myo‐inositol application were considered. The findings of 16 selected studies from 2240 citations indicated significant improvements in several parameters of sperm after Myo‐inositol administration. Myo‐inositol treatment was associated with a notable increase in total sperm motility (SMD 0.90; 95% CI: 0.34 to 1.46; *I* ^2^ = 0%, *p* = .001) and progressive sperm motility (SMD 1.48; 95% CI: 0.37 to 2.59; *I* ^2^  = 0%, *p* = .008). Additionally, there was a significant improvement in testosterone levels (SMD 0.54; 95% CI: 0.34 to 0.73; *I* ^2^  = 0%, *p* < .0001). Furthermore, Myo‐inositol therapy demonstrated a significant decrease in spermatozoa with DNA fragmentation (SMD −1.37; 95% CI: −2.43 to −0.32; *I* ^2^  = 85%, *p* = .01). This study suggests that Myo‐inositol therapy has a positive impact on specific sperm parameters, such as total and progressive sperm motility, along with testosterone levels. These findings provide support for the potential benefits of Myo‐inositol in improving male fertility parameters related to sperm factors.

## Graphical abstract

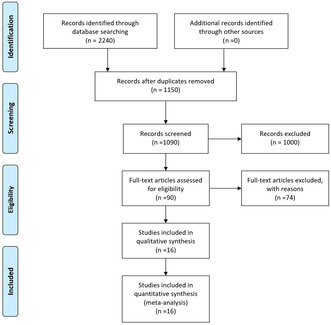

## 1 INTRODUCTION

About 40%–50% of infertility cases are due to male factors. It may be one or a combination of poor sperm motility (asthenospermia), low sperm concentration (oligospermia), and abnormal morphology (teratospermia), totally defined as oligoasthenospermia (OAS) (De Leo et al., 2022; Kumar & Singh, 2015). In vitro treatment of OAS semen samples with substances such as Myo‐inositol showed improved sperm quality, fertilization rate, and embryo quality (Korosi et al., 2017).

Myo‐inositol is a molecule that closely resembles sugar and is recognized as a member of the vitamin B family. It plays a crucial role in the composition of cell membranes, contributing to lipid synthesis and cellular signaling processes (Díaz et al., 2003). It plays a significant role in the reproduction process such as gametes and fetal development (Stachecki & Armant, 1996). Also, sperm motility, capacitation, maturation, and acrosome reaction were found modulated by Myo‐inositol (Chauvin & Griswold, 2004). Its antioxidant effect was demonstrated in male infertility treatment by optimizing sperm quality‐related parameters (Qamar et al., 2019; Vazquez‐Levin & Verón, 2020).

Given the potential of Myo‐inositol therapy to improve sperm quality and male fertility, but with conflicting results reported in previous studies, we conducted a systematic review and meta‐analysis to investigate the effects of Myo‐inositol on sperm factors and pregnancy rate. Specifically, we focused on the effects of Myo‐inositol in patients with oligo‐astheno‐teratozoospermia (OAT) who received either oral or in vitro supplementation. Our meta‐analysis evaluated multiple sperm parameters including sperm concentration, motility, morphology, viability, and DNA fragmentation.

## 2 METHODS

### 2.1 Search strategy

Following the guidelines outlined in the Cochrane Handbook, we performed a systematic review and meta‐analysis to investigate the research question at hand (Cumpston et al., 2019). The study protocol is registered at PROSPERO website with reference code CRD42023412506. We searched PubMed (Medline), Web of Science, and Embase systematically until February 20, 2023, using the search string: ((Inositol) OR (“Chiro‐Inositol”) OR (Mesoinositol) OR (Myoinositol)) AND ((Semen) OR (“Seminal Plasma”) OR (“Male infertility”) OR (“Male fertility”) OR (“Male subfertility”) OR (“Male sterility”) OR (“Male reproductive system”) OR (“Male reproduction”) OR (“Male reproductivity”) OR (Sperm) OR (Spermatozoa) OR (Spermatozoon) OR (Oligospermia) OR (Cryptospermia) OR (Cryptozoospermia) OR (Hypospermatogenesis) OR (Oligoasthenoteratozoospermia) OR (Oligozoospermia) OR (Azoospermia) OR (Aspermia) OR (“Sperm motility”) OR (“Sperm concentration”) OR (“Sperm morphology”) OR (“Sperm count”) OR (“total motile count”) OR (“Progressive motility”)). In addition, we examined the reference sections of other studies to identify relevant publications that were not present in the databases. The outcome was then transferred to the EndNote X9 software for additional evaluation.

### 2.2 Study selection and data extraction

Studies recognized as eligible for inclusion criteria: (1) in‐vivo/in‐vitro inositol therapy; (2) male participants diagnosed with infertility; (3) studies evaluating the effect of Myo‐inositol treatment on sperm factors and other outcomes relevant to this topic. We included randomized and non‐randomized clinical trials, observational studies (cross‐sectional, case–control, or cohort), and conference abstracts as a source of gray literature. Case reports, case series, non‐English articles, and studies that involved infertile couples without confirmed male factors or anatomical infertility factors were excluded.

The titles and abstracts, as well as the full texts in EndNote, were reviewed by two separate reviewers. Any differences or conflicts that arose were resolved through discussion with a third reviewer. The following data were extracted from text, tables, graphs, figures, and supplementary materials: author, year, country, type of study, population, number of participants, Myo‐inositol therapy details, duration of treatment (if applicable), ejaculate volume, Testosterone level, sperm factors (such as sperm concentration, progressive and total motility, normal morphology, viable spermatozoa, spermatozoa with DNA fragmentation), and pregnancy rate and data as IVF (In vitro fertilization), ICSI (intracytoplasmic sperm injection), and IUI (intrauterine insemination) (if applicable). A third reviewer resolved all disagreements.

### 2.3 Quality assessment

The included studies underwent independent evaluations by two reviewers using the NIH risk of bias checklists for Controlled Intervention Studies and Observational Cohort/Cross‐Sectional Studies (National Heart). Studies that obtained 11 or more affirmative responses were categorized as “Good,” those with 6 to 10 affirmative responses were classified as “Fair,” and studies with fewer than six affirmative responses were designated as “Poor.”

### 2.4 Outcome measure

The meta‐analysis's primary outcomes were total and progressive sperm motility, and secondary outcomes were testosterone level, sperm concentration, sperm normal morphology, viable spermatozoa, spermatozoa with DNA fragmentation, and pregnancy rate.

### 2.5 Statistical analysis

To report the relative outcomes, we utilized the standardized mean difference (MD) and its 95% confidence intervals (CI). The random effect model was utilized to combine the quantitative values from each study individually. In cases where a study did not present an effect size, an estimate was generated using Excel calculators. Heterogeneity was evaluated using Cochran's Q statistic (Q‐test) and the *I* ^2^ statistic. An *I* ^2^ value >75% indicated a high level of heterogeneity. Visual inspection of the funnel plot was used to evaluate publication bias for primary outcomes. Moderator analyses (study design and type of intervention used) were performed to see if any significant changes occur in the pooled effect size. The statistical analyses conducted in this study were all found to be statistically significant, with a *p*‐value of less than .05. These analyses were performed using R‐4.1.3 software and the Meta package, developed by the R Core Team in Vienna, Austria. The R software can be accessed at https://www.R‐project.org/.

## 3 RESULTS

### 3.1 Search results and characteristics

**FIGURE 1:**
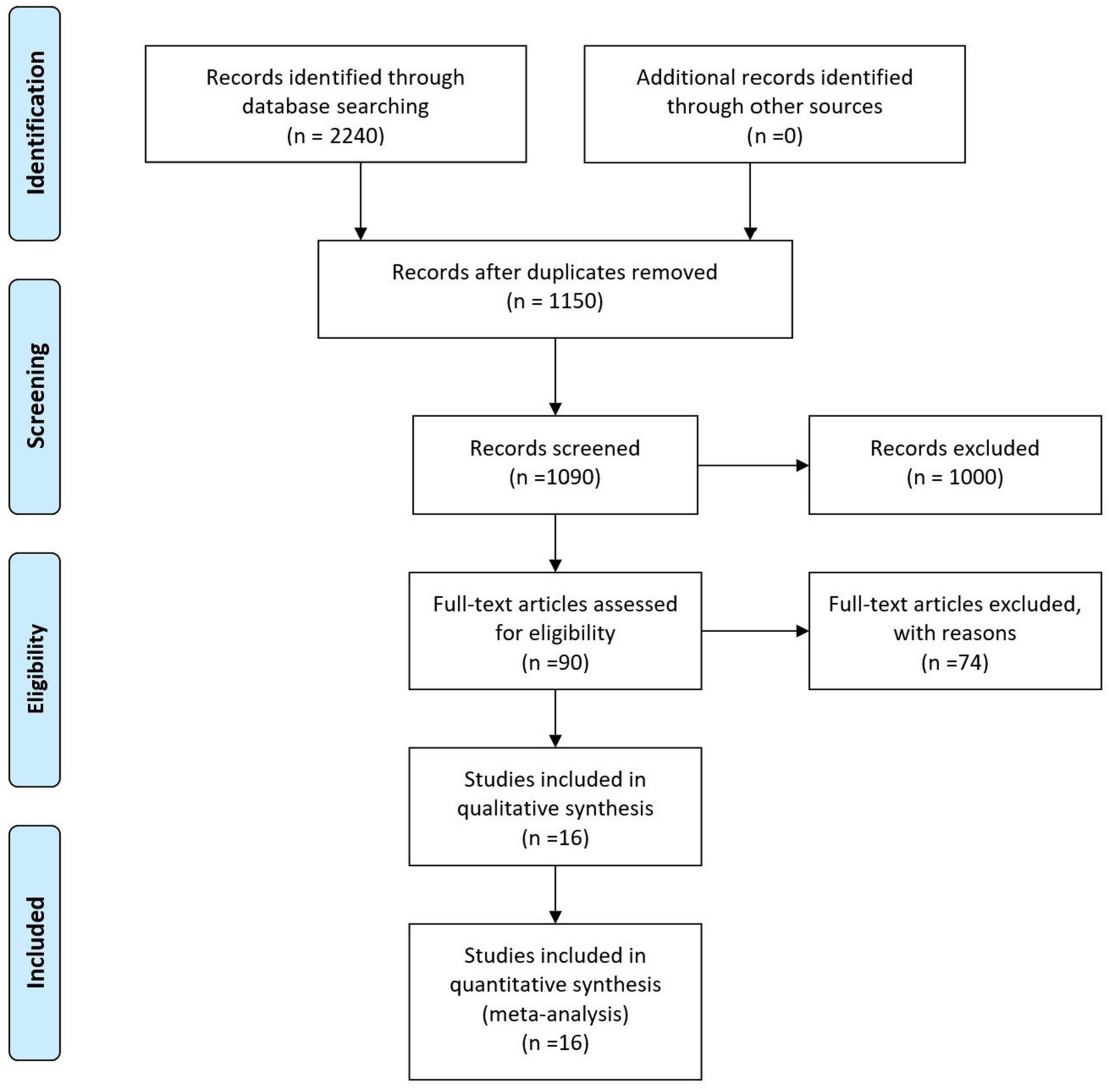
PRISMA flow diagram.

Out of a total of 2240 articles recorded through database searching, finally, 16 articles matched our eligibility criteria after full‐text review. After the removal of 1150 duplicates, studies were reviewed for meeting eligibility criteria. The study selection process is presented in the PRISMA flow diagram (Figure 1). Studies that were excluded were conducted in fertile male population, couples without exact male infertility factors, or lacked relevant information for analysis. The studies were conducted in different countries, including Italy [2, 3, 11–18], Bulgaria [19], Egypt [20, 21], and Iran [22–24]. The participants' ages ranged from 18 to 60 years. The studies were published between 2012 and 2022. Tables S3–S5 provide detailed characteristics of the included studies.

Among the studies included in the analysis, six were conducted as randomized clinical trials (RCTs) (Calogero et al., 2015; Canepa et al., 2018; Capece et al., 2017; Ghasemi et al., 2019; Korosi et al., 2017; Saleh et al., 2018). The duration of treatment varied significantly across the studies, with timeframes ranging from 30 min to 6 months. However, approximately half of the studies had treatment duration of around 3 months.

In all of the studies, the case group received MI at different doses. Moreover, three studies incorporated additional interventions for the case group, such as L‐carnitine, L‐arginine, vitamin E, and selenium (Dinkova et al., 2017; Korosi et al., 2017; Montanino Oliva et al., 2016). Another study included an intervention group that received a combination of L‐carnitine, acetyl L‐carnitine hydrochloride, vitamin E, vitamin C, coenzyme Q10, selenium, and vitamin D3 (De Leo et al., 2022).

### 3.2 Quality assessment

According to the NIH checklist, the quality assessment of the included studies was categorized as follows: Good (4 studies), Fair (12 studies), and none were classified as Poor. A comprehensive overview of the results from the quality assessment for each study can be found in the supplementary file (Figure S1).

## 4 RESULTS

### 4.1 Primary outcomes

The impact of MI on various sperm parameters, including sperm motility, sperm concentration, and the number of morphologically normal sperm, was assessed in seven studies (Canepa et al., 2018; Capece et al., 2017; Condorelli et al., 2012; De Leo et al., 2022; Ghasemi et al., 2019; Montanino Oliva et al., 2016; Saleh et al., 2018). In a study conducted by Azizi et al., the effects of MI (at a concentration of 2 mg/mL) on sperm concentration, the number of viable spermatozoa, total sperm motility, and progressive sperm motility were compared between semen specimens with MI, a control group, and fresh semen samples (Azizi et al., 2022). Four studies focused solely on evaluating the influence of MI treatment on sperm motility (Abdolsamadi et al., 2020; Artini et al., 2017; Dinkova et al., 2017; Palmieri et al., 2016; Saleh et al., 2017). Furthermore, Calogero et al. conducted a double‐blind RCT involving 194 patients with infertility of unknown cause. The case group received Inofolic, which consisted of 2 g MI and 200 mg folic acid, while the control group received a placebo. The study reported on the effects of MI on normal sperm morphology as well as total and progressive sperm motility (Calogero et al., 2015).

Most studies examining the effects of MI treatment on sperm motility have also reported its impact on progressive sperm motility, with the exception of four studies (Azizi et al., 2022; Dinkova et al., 2017; Ghasemi et al., 2019; Montanino Oliva et al., 2016). Additionally, Condorelli et al. conducted a comparative analysis using semen samples from 20 patients. The samples were treated with 2 mg/mL MI in vitro, while the control group was treated with saline. The researchers evaluated both progressive sperm motility and the number of viable spermatozoa in their assessment (Condorelli et al., 2012).

Furthermore, four studies specifically evaluated the effects of MI treatment on fertilization outcomes. In the study by Ghasemi et al. patients underwent intrauterine insemination (IUI) as a treatment for infertility (Ghasemi et al., 2019). Korosi et al. conducted a study using Physiological Intra‐Cytoplasmic Sperm Injection (PICSI) as the method of fertilization in their research (Korosi et al., 2017). De Leo et al. focused on patients undergoing in vitro fertilization (IVF) and examined the impact of MI treatment on the IVF process (De Leo et al., 2022).

#### 4.1.1 Total sperm motility

**FIGURE 2:**
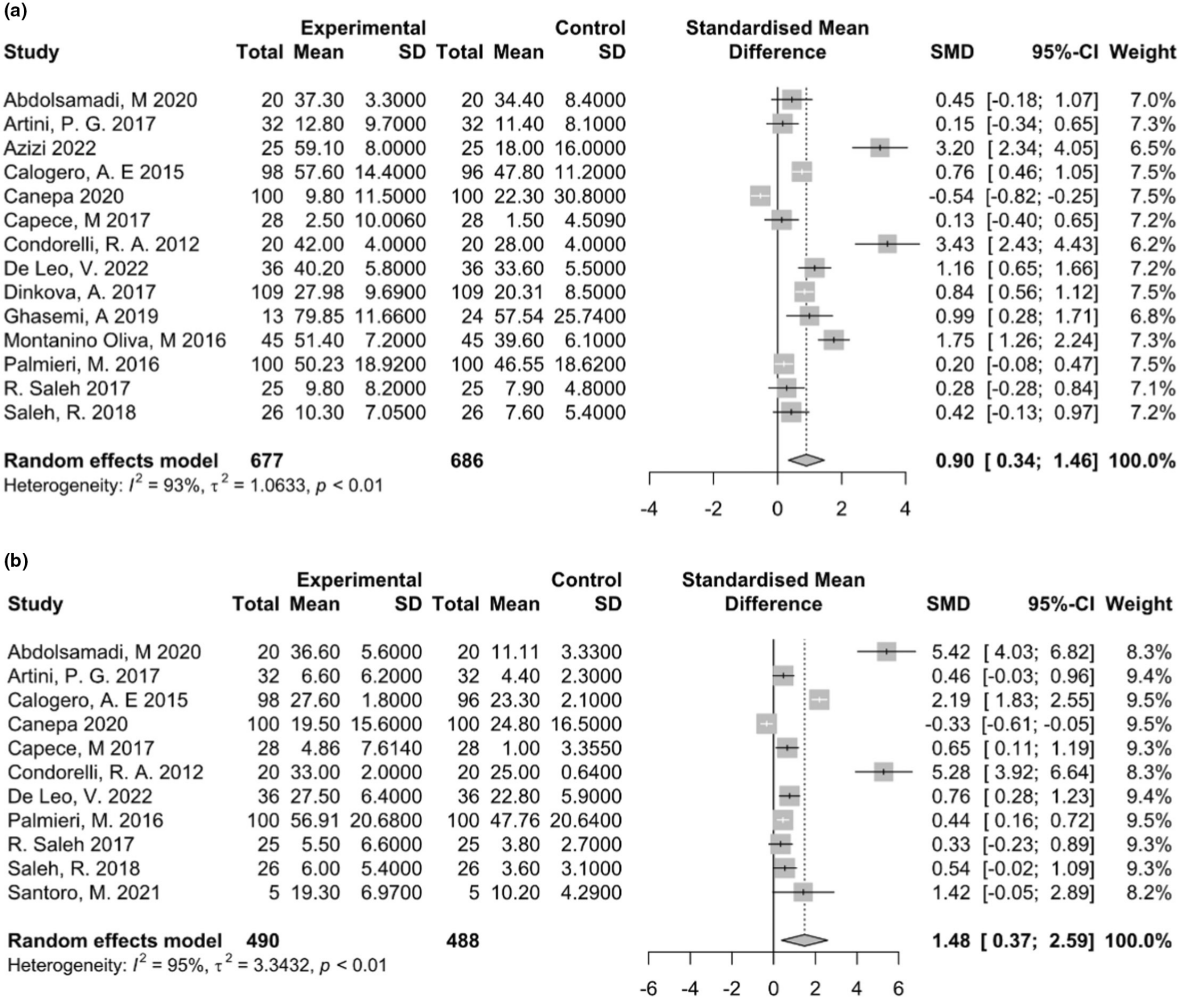
Results of meta‐analysis for (a) total sperm motility and (b) progressive sperm motility.

The results of our meta‐analysis of 14 studies showed a significant increase in total sperm motility in Myo‐inositol treated group (SMD 0.90; 95% CI: 0.34 to 1.46; *I* ^2^ = 0%, *p* = .001) (Figure 2a). Visual inspection of the funnel plot shows some sources of publication bias (Figure S2). Furthermore, our moderator analysis regarding study design (*p* = .89) and the type of intervention (Myo‐inositol alone or in combination) (*p* = .50) did not change the significance of our findings.

#### 4.1.2 Progressive sperm motility

The results of our meta‐analysis of 11 studies showed a significant increase in progressive sperm motility among Myo‐inositol treated group (SMD 1.48; 95% CI: 0.37 to 2.59; *I* ^2^ = 0%, *p* = .008) (Figure 2b). Visual inspection of the funnel plot shows some sources of publication bias (Figure S3). Furthermore, our moderator analysis regarding study design (*p* = .69) and the type of intervention (Myo‐inositol alone or in combination) (*p* = .37) did not change the significance of our findings.

### 4.2 Secondary outcomes

#### 4.2.1 Testosterone level

**FIGURE 3:**
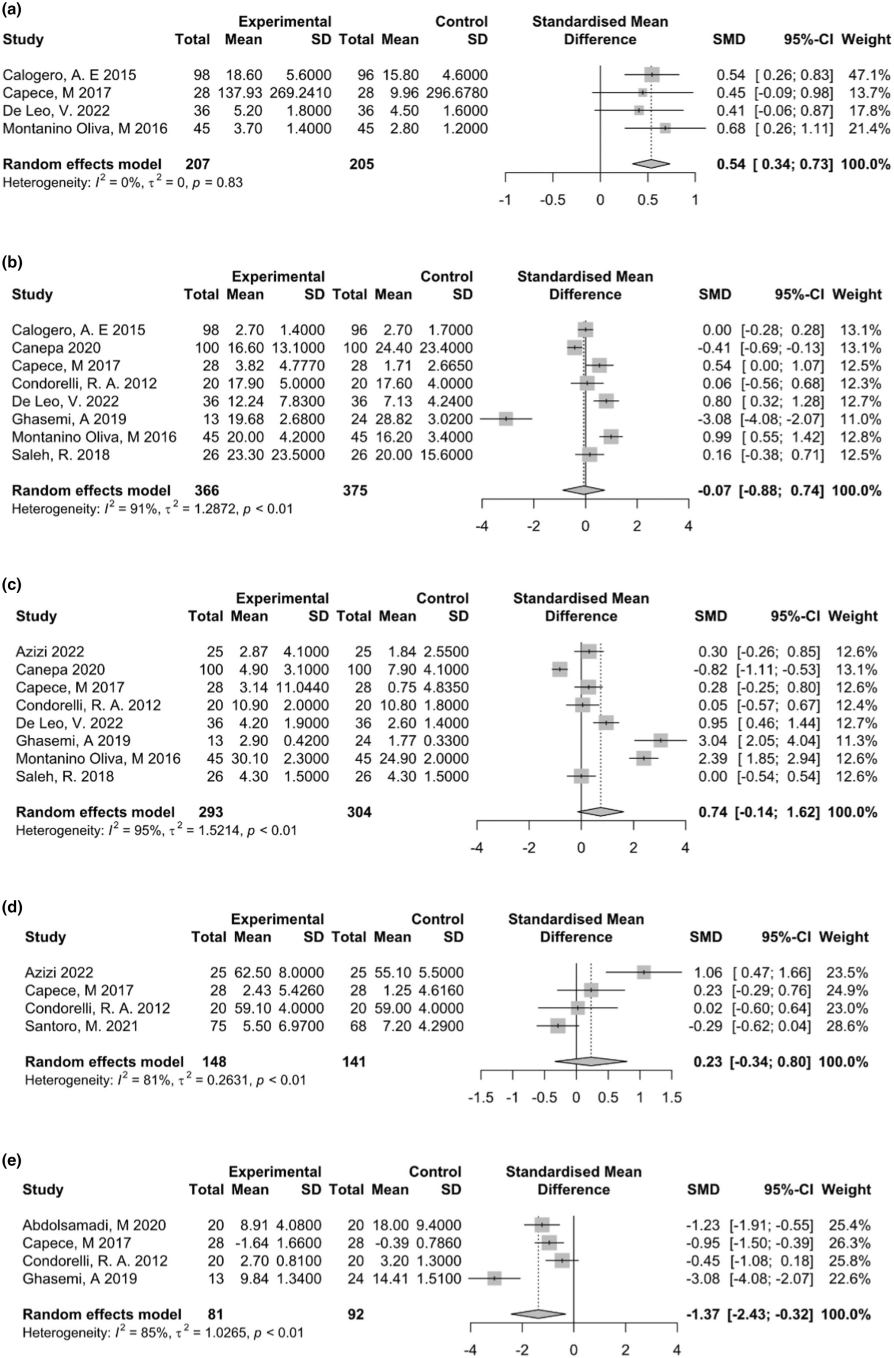
Results of meta‐analysis for (a) testosterone levels, (b) sperm concentration, (c) sperm normal morphology, (d) viable spermatozoa count, and (e) spermatozoa with DNA fragmentation.

Four studies have reported the impact of Myo‐inositol therapy on testosterone levels (Calogero et al., 2015; Capece et al., 2017; De Leo et al., 2022; Montanino Oliva et al., 2016). The results of our meta‐analysis showed a significant improvement in testosterone levels following Myo‐inositol therapy (SMD 0.54; 95% CI: 0.34 to 0.73; *I* ^2^ = 0%, *p* < .0001) (Figure 3a).

#### 4.2.2 Sperm concentration

The results of our meta‐analysis of eight studies showed no significant change in sperm concentration following Myo‐inositol therapy (SMD −0.07; 95% CI: −0.88 to 0.74; *I* ^2^ = 91%, *p* = .86) (Figure 3b).

#### 4.2.3 Sperm normal morphology

The results of our meta‐analysis of eight studies showed a non‐significant increase in sperm normal morphology following Myo‐inositol therapy (SMD 0.74; 95% CI: −0.14 to 1.62; *I* ^2^ = 95%, *p* = .1) (Figure 3c).

#### 4.2.4 Viable spermatozoa

The results of our meta‐analysis of four studies showed a non‐significant increase in sperm normal morphology following Myo‐inositol therapy (SMD 0.23; 95% CI: −0.34 to 0.80; *I* ^2^ = 81%, *p* = .42) (Figure 3d).

#### 4.2.5 Spermatozoa with DNA fragmentation

The results of our meta‐analysis showed a significant decrease in spermatozoa with DNA fragmentation following Myo‐inositol therapy (SMD −1.37; 95% CI: −2.43 to −0.32; *I* ^2^ = 85%, *p* = .01) (Figure 3e).

#### 4.2.6 Pregnancy rate

**FIGURE 4:**
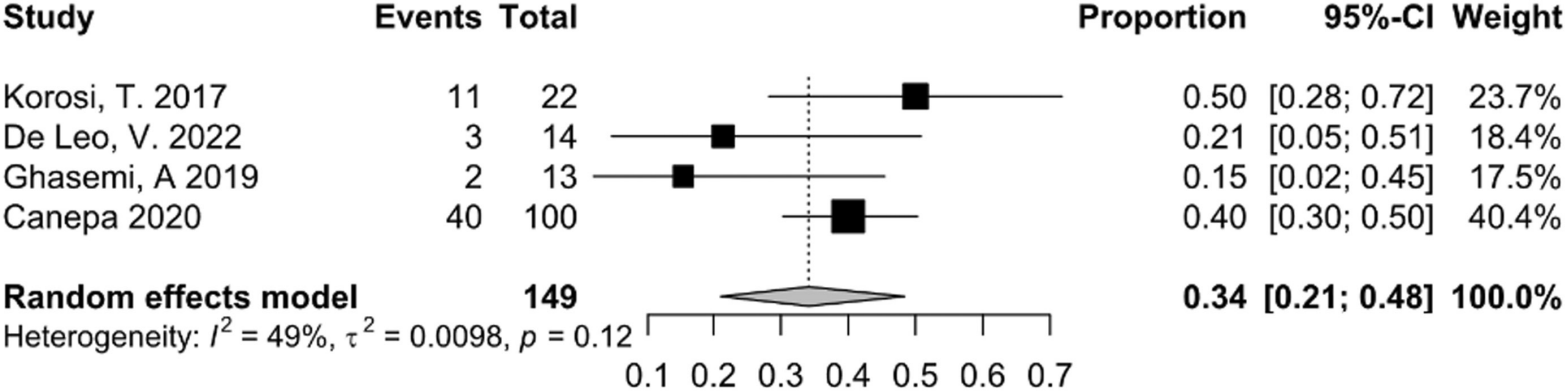
Meta‐analysis for pregnancy rate among Myo‐inositol treated group.

By pooling their effect sizes regarding the pregnancy rate in the Myo‐inositol receiving group, the rate of pregnancy was 34% (95% CI: 21% to 48%) (Figure 4).

## 5 DISCUSSION

The present meta‐analysis aimed to assess the impact of Myo‐inositol therapy on various sperm parameters according to WHO criteria (Cooper et al., 2010). The findings indicate a significant increase in total sperm motility and progressive sperm motility among the Myo‐inositol‐treated group. These results suggest a favorable effect of Myo‐inositol therapy on sperm motility parameters. The high heterogeneity observed in both analyses indicates differences in the design and characteristics of included studies. However, potential publication bias was noted through visual inspection of the funnel plots, emphasizing the need for cautious interpretation. Moderator analyses based on study design and the type of intervention did not influence the significance of the results.

Sperm motility is an essential factor for male fertility, both in natural conception and assisted reproduction (Dcunha et al., 2022; Donnelly et al., 1998; Hirano et al., 2001; Marchetti et al., 2002). Several mechanisms have been proposed to explain the effect of Myo‐inositol on sperm motility. Myo‐inositol may reduce mitochondrial cristae damage, which is present in patients with oligo‐asthenospermia, leading to improvements in mitochondrial membrane potential and sperm motility (Colone et al., 2010; Condorelli et al., 2011, 2012; Marchetti et al., 2002; Wang et al., 2003). Additionally, the activation of phospholipase C enzymes by Myo‐inositol could increase intracellular and mitochondrial Ca^2+^ ion concentrations, subsequently regulating ATP (Adenosine Triphosphate) synthesis and enhancing total sperm motility (Condorelli et al., 2017; Shikh et al., 2022).

In terms of testosterone levels, our analysis revealed a significant improvement following Myo‐inositol therapy. These findings suggest that Myo‐inositol may positively impact testosterone levels. No heterogeneity was observed among the included studies, indicating consistency in the results. It should be noted that although testosterone is needed for spermatogenesis, low testosterone levels seem to be sufficient for spermatogenesis, as demonstrated in a relatively large cohort study of 853 infertile men, testosterone level does not seem to affect sperm characteristics (Di Guardo et al., 2020).

There was no significant change in sperm concentration following Myo‐inositol therapy, indicating that Myo‐inositol may not directly influence this parameter. The high heterogeneity observed in this analysis should be considered when interpreting the results.

Regarding sperm morphology, our analysis revealed a non‐significant increase in sperm normal morphology following Myo‐inositol therapy. While the trend suggested improvement, it is important to consider the lack of statistical significance and high heterogeneity. Further studies are needed to explore the molecular mechanisms that influence sperm morphology (Vazquez‐Levin & Verón, 2020).

The analysis did not find a significant increase in viable spermatozoa count following Myo‐inositol therapy. These findings suggest that Myo‐inositol may not have a substantial impact on the number of viable spermatozoa. The moderate heterogeneity observed in this analysis indicates some variability among the included studies.

On the other hand, this meta‐analysis revealed a significant decrease in spermatozoa with DNA fragmentation following Myo‐inositol therapy. This suggests a beneficial effect of Myo‐inositol in reducing DNA fragmentation, which is important for sperm DNA integrity. Moderate heterogeneity was observed in this analysis, indicating some variability among the studies.

Sperm DNA integrity is influenced by both testicular and post‐testicular mechanisms (A. Agarwal et al., 2020). One of the suggested mechanisms that affect DNA integrity and male fertility is oxidative stress (Bisht et al., 2017). The imbalance between reactive oxygen species and endogenous antioxidants leads to oxidative stress, which can affect multiple sperm characteristics, including DNA fragmentation (Agarwal et al., 2009, 2014; Zandieh et al., 2018). Myo‐inositol has been shown to reduce inflammation and oxidative stress, potentially playing a protective role (Baldassarre et al., 2021).

Four studies included in our analysis reported pregnancy rates in addition to sperm parameters. By pooling the effect sizes, the meta‐analysis revealed a pregnancy rate of 34% (95% CI: 21% to 48%) in the Myo‐inositol receiving group. However, it should be noted that the number of studies reporting pregnancy outcomes was limited. Therefore, further research with a larger sample size is needed to establish a more comprehensive understanding of the relationship between Myo‐inositol therapy and pregnancy rates.

Myo‐inositol impact on male fertility is not only limited to the mentioned factors. Obesity is a well‐known risk factor for male infertility (Katib, 2015). A systematic review and meta‐analysis by Zarezadeh et al. reported that Myo‐inositol supplements have a significant effect on Body Mass Index (BMI) reduction (Zarezadeh et al., 2022). Another meta‐analysis showed that Myo‐inositol could also improve glucose metabolism and insulin resistance which is another risk factor of male infertility (Miñambres et al., 2019; Zańko et al., 2022). These findings add to the clinical benefit of adding Myo‐inositol to the drug regimen of infertile men.

Subsequent investigations should focus on tackling the limitations discovered in this meta‐analysis. These limitations include incorporating larger sample sizes, implementing standardized treatment protocols, and conducting a comprehensive evaluation of fertility outcomes. Moreover, further research is essential to enhance our understanding of the underlying mechanisms through which Myo‐inositol influences sperm parameters and fertility.

Several limitations should be acknowledged in this meta‐analysis. Firstly, the quality and design of the included studies varied which may introduce bias and affect the overall robustness of the results. Secondly, the presence of potential publication bias, as indicated by the funnel plots, suggests that studies with positive findings may have been more likely to be published, leading to an overestimation of the treatment effect (Nair, 2019). In addition, the heterogeneity observed in analyses may be attributed to differences in study characteristics, such as sample size, study population, and treatment protocols. Finally, the limited number of studies reporting pregnancy outcomes restricts the ability to draw definitive conclusions regarding the clinical impact of Myo‐inositol therapy on fertility. To the best of our knowledge, this meta‐analysis is the first study to investigate the impact of Myo‐inositol on male fertility, although further studies are needed, the result of the present study showed that Myo‐inositol could improve sperm quality and its biochemical factor.

## 6 CONCLUSION

In conclusion, the findings of this meta‐analysis suggest that Myo‐inositol therapy has a positive impact on certain sperm parameters, including total and progressive sperm motility, as well as testosterone levels. These results indicate the potential benefits of Myo‐inositol in improving male fertility. However, no significant changes were observed in sperm concentration, sperm normal morphology, viable spermatozoa count, and pregnancy rates. The reduction in spermatozoa with DNA fragmentation suggests a potential protective effect of Myo‐inositol therapy on sperm DNA integrity. These results recommend Myo‐inositol as a potential treatment for male infertility and shed light on the possibility of using Myo‐inositol as an accessible and safe treatment by healthcare providers. Nevertheless, it is important to consider the potential publication bias and the limitations of the included studies. Further research with larger sample sizes is warranted to validate these findings and explore the clinical implications of Myo‐inositol therapy on fertility outcomes.

## AUTHOR CONTRIBUTIONS

**Marjan Ghaemi:** Conceptualization (equal). **Niloofar Seighali:** Investigation (equal); methodology (equal). **Zahra Panahi:** Methodology (equal); writing – review and editing (equal). **Arman Shafiee:** Methodology (equal); writing – review and editing (equal). **Maryam Beiky:** writing – review and editing (equal). **Omid Kohandel Gargari:** writing – review and editing (equal). **Alireza Azarboo:** Investigation (equal). **Vida Shafti:** Investigation (equal). **Kyana Jafarabady:** Methodology (equal); writing – review and editing (equal). **Nasim Eshraghi:** Conceptualization (equal). **Mohammad Haddadi:** writing – review and editing (equal). **Razieh Akbari:** writing – review and editing (equal). **Sedigheh Hantoushzadeh:** Conceptualization (equal); Supervision (equal).

## FUNDING INFORMATION

The authors declare no funding information.

## CONFLICT OF INTEREST STATEMENT

The authors have no conflict of interest to declare.

## Supporting information


Data S1:

## Data Availability

Data sharing is available by contacting the corresponding author.
