# Supplementary material for: The effect of Myo‐inositol on improving sperm quality and IVF outcomes: A systematic review and meta‐analysis
Source: Food Sci Nutr. 2024 Sep 12;12(11):8515–24. doi: 10.1002/fsn3.4427 (PMC11606893; doi:10.1002/fsn3.4427)

# Supplementary Material

**The effect of Myo-inositol therapy on sperm quality and its biochemical factors: a systematic review and meta-analysis.**

**Supplementary Table 1-** PRISMA 2020 checklist

**Supplementary Table 2-** Search strategies for online databases

**Supplementary Table 3-** Characteristics of the included studies

**Supplementary Table 4-** Results of the included studies (Sperm parameters)

**Supplementary Table 5-** Pregnancy data of the studies

**Supplementary Figure 1-** Risk of bias assessment for each included study

**Supplementary Figure 2-** Funnel plot for total sperm motility

**Supplementary Figure 3-** Funnel plot for progressive sperm motility

This supplemental material has been provided by the authors to give readers additional information about their work.

**Supplementary Table 1:** PRISMA 2020 checklist

| **Section and Topic** | **Item #** | **Checklist item** | **Reported on page #** |
| --- | --- | --- | --- |
| **TITLE** | | |  |
| Title | 1 | Identify the report as a systematic review. | 1 |
| **ABSTRACT** | | |  |
| Abstract | 2 | See the PRISMA 2020 for Abstracts checklist. | 3, 4 |
| **INTRODUCTION** | | |  |
| Rationale | 3 | Describe the rationale for the review in the context of existing knowledge. | 5 |
| Objectives | 4 | Provide an explicit statement of the objective(s) or question(s) the review addresses. | 6 |
| **METHODS** | | |  |
| Eligibility criteria | 5 | Specify the inclusion and exclusion criteria for the review and how studies were grouped for the syntheses. | 6, 7 |
| Information sources | 6 | Specify all databases, registers, websites, organisations, reference lists and other sources searched or consulted to identify studies. Specify the date when each source was last searched or consulted. | 6 |
| Search strategy | 7 | Present the full search strategies for all databases, registers and websites, including any filters and limits used. | Suppl. |
| Selection process | 8 | Specify the methods used to decide whether a study met the inclusion criteria of the review, including how many reviewers screened each record and each report retrieved, whether they worked independently, and if applicable, details of automation tools used in the process. | 7 |
| Data collection process | 9 | Specify the methods used to collect data from reports, including how many reviewers collected data from each report, whether they worked independently, any processes for obtaining or confirming data from study investigators, and if applicable, details of automation tools used in the process. | 7, 8 |
| Data items | 10a | List and define all outcomes for which data were sought. Specify whether all results that were compatible with each outcome domain in each study were sought (e.g. for all measures, time points, analyses), and if not, the methods used to decide which results to collect. | 7 |
|  | 10b | List and define all other variables for which data were sought (e.g. participant and intervention characteristics, funding sources). Describe any assumptions made about any missing or unclear information. | 7 |
| Study risk of bias assessment | 11 | Specify the methods used to assess risk of bias in the included studies, including details of the tool(s) used, how many reviewers assessed each study and whether they worked independently, and if applicable, details of automation tools used in the process. | 8 |
| Effect measures | 12 | Specify for each outcome the effect measure(s) (e.g. risk ratio, mean difference) used in the synthesis or presentation of results. | 8 |
| Synthesis methods | 13a | Describe the processes used to decide which studies were eligible for each synthesis (e.g. tabulating the study intervention characteristics and comparing against the planned groups for each synthesis (item #5)). | 8 |
|  | 13b | Describe any methods required to prepare the data for presentation or synthesis, such as handling of missing summary statistics, or data conversions. | 8 |
|  | 13c | Describe any methods used to tabulate or visually display results of individual studies and syntheses. | 8 |
|  | 13d | Describe any methods used to synthesize results and provide a rationale for the choice(s). If meta-analysis was performed, describe the model(s), method(s) to identify the presence and extent of statistical heterogeneity, and software package(s) used. | 8 |
|  | 13e | Describe any methods used to explore possible causes of heterogeneity among study results (e.g. subgroup analysis, meta-regression). | 9 |
|  | 13f | Describe any sensitivity analyses conducted to assess robustness of the synthesized results. | 9 |
| Reporting bias assessment | 14 | Describe any methods used to assess risk of bias due to missing results in a synthesis (arising from reporting biases). | 8 |
| Certainty assessment | 15 | Describe any methods used to assess certainty (or confidence) in the body of evidence for an outcome. | 9 |
| **RESULTS** | | |  |
| Study selection | 16a | Describe the results of the search and selection process, from the number of records identified in the search to the number of studies included in the review, ideally using a flow diagram. | 10, Figure 1 |
|  | 16b | Cite studies that might appear to meet the inclusion criteria, but which were excluded, and explain why they were excluded. | Figure 1 |
| Study characteristics | 17 | Cite each included study and present its characteristics. | 10, suppl. |
| Risk of bias in studies | 18 | Present assessments of risk of bias for each included study. | 10, suppl. |
| Results of individual studies | 19 | For all outcomes, present, for each study: (a) summary statistics for each group (where appropriate) and (b) an effect estimate and its precision (e.g. confidence/credible interval), ideally using structured tables or plots. | Figures, suppl. |
| Results of syntheses | 20a | For each synthesis, briefly summarise the characteristics and risk of bias among contributing studies. | 10-12 |
|  | 20b | Present results of all statistical syntheses conducted. If meta-analysis was done, present for each the summary estimate and its precision (e.g. confidence/credible interval) and measures of statistical heterogeneity. If comparing groups, describe the direction of the effect. | 10-12 |
|  | 20c | Present results of all investigations of possible causes of heterogeneity among study results. | 11, 12, Table 1 |
|  | 20d | Present results of all sensitivity analyses conducted to assess the robustness of the synthesized results. | 11 |
| Reporting biases | 21 | Present assessments of risk of bias due to missing results (arising from reporting biases) for each synthesis assessed. | 11, 12 |
| Certainty of evidence | 22 | Present assessments of certainty (or confidence) in the body of evidence for each outcome assessed. | 11, 12, suppl. |
| **DISCUSSION** | | |  |
| Discussion | 23a | Provide a general interpretation of the results in the context of other evidence. | 12-14 |
|  | 23b | Discuss any limitations of the evidence included in the review. | 14 |
|  | 23c | Discuss any limitations of the review processes used. | 14 |
|  | 23d | Discuss implications of the results for practice, policy, and future research. | 14, 15 |

**Supplementary Table 2:** Search strategies for online databases

| **MEDLINE (via PubMed)** | 1.“Inositol” [mh] OR Inositol[tiab] OR Chiro-Inositol[tiab] OR Mesoinositol[tiab] OR Myoinositol[tiab]  2- “Semen” [mh] OR “Spermatozoa” [mh] OR Semen [tiab] OR Seminal [tiab] OR Male infertility[tiab] OR Male fertility[tiab] OR Male subfertility[tiab] OR Male sterility[tiab] OR Male reproductive system[tiab] OR Male reproduction[tiab] OR Male reproductivity[tiab] OR Sperm[tiab] OR Spermatozoa [tiab] OR Spermatozoon[tiab] OR Oligospermia[tiab] OR Cryptospermia[tiab] OR Cryptozoospermia[tiab] OR Hypospermatogenesis[tiab] OR Oligoasthenoteratozoospermia[tiab] OR Oligozoospermia[tiab] OR Azoospermia[tiab] OR Aspermia[tiab] OR total motile count[tiab] OR Progressive motility[tiab]  3- #1 AND #2 |
| --- | --- |
| **Embase** | 1- ‘Inositol’/exp OR ‘Inositol’:ti,ab OR ‘Chiro-Inositol’:ti,ab OR ‘Mesoinositol’:ti,ab OR ‘Myoinositol’:ti,ab  2- ‘Sperm’/exp OR ‘spermatozoon’/exp OR ‘Semen ‘:ti,ab OR ‘Seminal ‘:ti,ab OR ‘Male infertility’:ti,ab OR ‘Male fertility’:ti,ab OR ‘Male subfertility’:ti,ab OR ‘Male sterility’:ti,ab OR ‘Male reproductive system’:ti,ab OR ‘Male reproduction’:ti,ab OR ‘Male reproductivity’:ti,ab OR ‘Sperm’:ti,ab OR ‘Spermatozoa ‘:ti,ab OR ‘Spermatozoon’:ti,ab OR ‘Oligospermia’:ti,ab OR ‘Cryptospermia’:ti,ab OR ‘Cryptozoospermia’:ti,ab OR ‘Hypospermatogenesis’:ti,ab OR ‘Oligoasthenoteratozoospermia’:ti,ab OR ‘Oligozoospermia’:ti,ab OR ‘Azoospermia’:ti,ab OR ‘Aspermia’:ti,ab OR ‘total motile count’:ti,ab OR ‘Progressive motility’:ti,ab  3- #1 AND #2 |
| **Web of Science** | 1- “Inositol “OR “Chiro-Inositol” OR “Mesoinositol” OR “Myoinositol”  2- “Semen “ OR “Seminal “ OR “Male infertility” OR “Male fertility” OR “Male subfertility” OR “Male sterility” OR “Male reproductive system” OR “Male reproduction” OR “Male reproductivity” OR “Sperm” OR “Spermatozoa “ OR “Spermatozoon” OR “Oligospermia” OR “Cryptospermia” OR “Cryptozoospermia” OR “Hypospermatogenesis” OR “Oligoasthenoteratozoospermia” OR “Oligozoospermia” OR “Azoospermia” OR “Aspermia” OR “total motile count” OR “Progressive motility”  3- #1 AND #2 |

**Supplementary Table 3:** Characteristics of the included studies

| **Author, Year** | **Country** | **Type of study** | **Population** | **Total number of patients, n** | **Myoinositol (MI) therapy details** | **Duration of treatment** |
| --- | --- | --- | --- | --- | --- | --- |
| Condorelli et al., 2012 | Italy | in-vitro | Oligoastenospermia | 20 | 2 mg/mL | 2 hours |
| Calogero et al., 2015 | Italy | Randomized, placebo-controlled | Idiopathic infertility | 194 | Group 1: Inofolic ( 2 g of MI and 200 lg of folic acid) twice daily Group 2: placebo(folic acid alone) twice daily | 3 months |
| Montanino et al., 2016 | Italy | Prospective longitudinal | Asthenospermia with multiple sclerosis | 45 | 1g MI, 30mg L-carnitine, L-arginine and vitamin E, 55𝜇g selenium, and 200 𝜇g folic acid / twice a day | 3 months |
| Palmieri et al., 2016 | Italy | in-vitro | Oligozoospermia, Asthenozoospermia, Oligoasthenozoospermia | 19 oligozoospermic  15 asthenozoospermic  20 oligoasthenozoospermic 25 thawed semen samples | 100 μl | NA |
| Capece et al., 2017 | Italy | Randomized, placebo controlled | Oligoastenospermia | 60 | group A : MI 1000 mg, Tribulus Terrestris 300 mg, Alga Ecklonia Bicyclis 200 mg and Biovis, one tablet a day group B : One placebo tablet a day | 3 months |
| [1]Dinkova et al., 2017 | Bulgaria | Prospective longitudinal | Asthenozoospermia | 109 | 1 g of MI, 30 mg of L-carnitine, L-arginine and Vitamin E, 55 μg of selenium, and 200 μg of folic acid , taken twice a day. | 3 months |
| Saleh et al., 2017 | Egypt | Randomized, placebo controlled | Infertile men | 25 | 1 mg divided in 2 aliquots (0.5 ml each) for grop A and B:  Case group (A): treated with cryo-protectant plus 10 ml MI solution Control group (B): treated with cryo-protectant alone | 6 months |
| Artini et al., 2017 | Italy | in-vitro | Oligoastenospermia | 32 | 15 ml/ml | 30 min |
| Saleh et al., 2018 | Egypt | in-vitro | Infertile men | 26 | MI :0.5 mL of semen with 0.5 mL of sperm freezing medium + a dose of 10 μL/mL of MI CONTROL : 0.5 mL of semen with 0.5 mL of sperm freezing medium + 10 μL/mL of sperm washing and preparation medium | NA |
| Ghasemi et al., 2019 | Iran | Randomized, placebo controlled | oligoasthenospermia or asthenospermia | 37 | Case group= 0.5 ml of MI with a concentration of 2 mg/ml and incubated at 37°C for 2 hr Control group= no interventions, incubated for 2 hr at 37°C in 0.5 mL of the flushing medium in the special kit | 2 hours |
| Canepa et al., 2020 | Italy | Randomized, placebo controlled | oligospermia and/or asthenospermia | 100 | 2 tablets/day of Sinopol : contained ALA (800 mg), MI (1000 mg), folic acid (400 mg), betaine (100 mg), vitamin B2 (1.7 mg), B6 (1.9 mg), and B12 (2.6 mg) | 3 months |
| Abdolsamadi et al., 2020 | Iran | in-vitro | Oligoastenospermia | 40 | 2 ml/ml | 1 month |
| Santoro et al., 2021 | Italy | Prospective longitudinal | Oligoastenospermia | 30 | MI 2 mg/ml, 30min | 3 months |
| Azizi et al., 2022 | Iran | in-vitro | Asthenospermia | 25 | 1, 2, 3 mg/ml Each semen sample was randomly divided into three groups: Fresh, MI (with freezing medium and 2mg/ml MI), and Control (with freezing medium alone) | NA |
| De Leo et al., 2022 | Italy | Prospective longitudinal | Oligoastenospermia | 36 | Inositol (1000 mg) , L-Carnitine(250 mg ), Acetyl L-Carnitine Hydrochloride(250 mg) , Vitamin E(60 mg) , Vitamin C(100 mg) , Coenzyme Q10(20 mg) , Selenium(50 mcg) , Vitamin D3(5 mcg)/ Daily | 3 months |
| Korosi et al., 2017 | Italy | Randomized, placebo controlled | Oligoastenospermia | 22 | 1 g of MI, 30 mg of L-carnitine, L-arginine and Vitamin E, 55 μg of selenium, and 200 μg of folic acid (Folandrol®, Exeltis, Hungary) twice a day [samples of the treated group = incubation for 2 h with 2 mg/ml of MI dispersed in the in vitro fertilization medium] Control group (n=13): No treatment in the same time | 2 months |

**Supplementary Table 4:** Results of the included studies (Sperm parameters)

| **Author, Year** | **Testosterone level (ng/ml) (Mean ± SD)** | **Sperm concentration (Mil/ml) (Mean ± SD)** | **Sperm total motility (%) (Mean ± SD)** | **Sperm progressive motility (%) (Mean ± SD)** | **Sperm normal morphology (%) (Mean ± SD)** | **Seminal leukocytes (Mil/ml) (Mean ± SD)** | **Viable spermatozoa (%) (Mean ± SD)(106/ml)** | **Spermatozoa with DNA fragmentation (%) (Mean ± SD)** |
| --- | --- | --- | --- | --- | --- | --- | --- | --- |
| Condorelli et al., 2012 | NA | Before treatment: 17.6 ± 4.0 After treatment: 17.9 ± 5.0 | Before treatment: 28.0 ± 4.0 After treatment: 42.0 ± 4.0 | Before treatment: 25.0 ± 0.64 After treatment: 33.0 ± 2.0 | Before treatment: 10.8 ± 1.8 After treatment: 10.9 ± 2.0 | Before treatment: 0.2 ± 0.2 After treatment: 0.4 ± 0.2 | Before treatment: 59.0 ± 4.0 After treatment: 59.1 ± 4.0 | Before treatment: 3.2 ± 1.3 After treatment: 2.70 ± 0.81 |
| Calogero et al., 2015 | Before treatment: group 1 before treatment : 15.8 ± 5.4 group 2 before treatment :15.6 ± 4.8 After treatment : group 1 : 18.6 ± 5.6 group 2 : 15.8 ± 4.6 | Before treatment: group 1 : 2.7 ± 1.3 group 2 :2.8 ± 1.4 After treatment: group 1 : 2.7 ± 1.4 group 2 : 2.7 ± 1.7 | Before treatment: group 1 : 22.2 ± 2.1 group : 22.3 ± 2.6 After treatment: group 1 : 27.6 ± 1.8 group 2 : 23.3 ± 2.1 | Before treatment: group 1: 22.2 2.1 group 2: 22.3 2.6 After treatment: group 1: 27.6 1.8 group 2: 23.3 2.1 | NA | NA | NA | NA |
| Montanino et al., 2016 | Before treatment: Total : 2.8 ± 1.2, Free : 33.0 ± 11.1 After treatment: Total : 3.7 ± 1.4, Free : 47.2 ± 13.0 | Before treatment: 16.2±3.4 After treatment: 20 ± 4.2 | Before treatment: 39.6±6.1 After treatment: 51.4 ± 7.2 | NA | Before treatment: 24.9 ± 2.0 After treatment: 30.1 ± 2.3 | NA | NA | NA |
| Palmieri et al., 2016 | NA | NA | Before treatment: [fresh samples before capacitation : 46.55 ± 18.62%/ after capacitation: 73.99 ± 28.94%. Thawed : 11.4 ± 16.51%] After treatment: [fresh samples before capacitation: 50.23 ± 18.92%/after capacitation : 70.87 ± 31.46%.      thawed : 14.88 ± 16.86%] | Before treatment: [before capacitation :47.76 ± 20.64% /after capacitation: 70.67 ± 26.72%.  Thawed: 9.8 ± 14.1%] After treatment: [ before capacitation : 56.91 ± 20.68%/after capacitation: 69.97 ± 27.27%  thawed: 16.4 ± 20.64%] | NA | NA | NA | NA |
| Capece et al., 2017 | Before treatment:Group A : 536.8 ± 190.5 Group B : 2573.1 ± 203.1 After treatment: Group A : 137.93 ± 269.241 Group B :9.96 ± 296.678 | Before treatment:Group A : 16.1 ± 1.9 Group B : 16.3 ± 2.0 After treatment: Group A : 3.82 ± 4.777 Group B :1.71 ± 2.665 | Before treatment: Group A : 27 ± 6.0 Group B : 26.5 ± 5.2 After treatment: Group A : 2.50 ± 10.006 Group B :1.50 ± 4.509 | Before treatment: Group A : 22.3 ± 4.8 Group B :22.8 ± 4.2 After treatment: Group A : 4.86 ± 7.614 Group B : 1.00 ± 3.355 | Before treatment: Group A : 15.4 ± 7 Group B : 13.9 ± 7.1 After treatment: Group A : 3.14 ± 11.044 Group B :0.75 ± 4.835 | Before treatment: Group A : 0.4 ± 0.2 Group B :0.4 ± 0.2 After treatment: Group A : 0.146 ± 0.3061 Group B : 0.025 ± 0.1531 | Before treatment: Group A : 53.4 ± 3.7 Group B :54.3 ± 4 After treatment: Group A : 2.43 ± 5.426 Group B :1.25 ± 4.616 | Before treatment: Group A : 4.8 ± 1.5 Group B : 5.2 ± 1.2 After treatment: Group A : 1.64 ± 1.660 Group B : 0.39 ± 0.786 |
| Dinkova et al., 2017 | NA | NA | Before treatment: 20.31% (SD ± 8.5) After treatment: 27.98 (SD ± 9.69) | NA | NA | NA | NA | NA |
| Saleh et al., 2017 | NA | NA | Before treatment: [PRE-FREEZ: 40 (31, 45)] After treatment: [Group A: 10 (4.5, 15), Group B :10 (3.8, 10)] | Before treatment: [PRE-FREEZ: 25 (16, 25)] After treatment: [Group A: 5 (1.5, 10), Group B : 5 (1.8, 5)] | NA | NA | NA | NA |
| Artini et al., 2017 | NA | 27.4 ± 20.1 | Before treatment: 11.4 ± 8.1 After treatment: 12.8 ± 9.7 | Before treatment: 4.4 ± 2.3 After treatment: 6.6 ± 6.2 | NA | NA | NA | NA |
| Saleh et al., 2018 | NA | Prefreez:46.6 ± 23.5 post thaw: [ MI group= 23.3 ± 23.5, Control group= 20 ± 15.6] | Prefreez: 40 ± 15.6 post thaw: [ MI group=10.3 ± 7.05, Control group= 7.6 ± 5.4 ] | Prefreez: 20.33 ± 19.60 post thaw: [ MI group= 6 ± 5.4 , Control group=3.6 ± 3.1 ] | Prefreez: 4.3 ± 1.5  post thaw: [ MI group= 4.3 ± 1.5, Control group= 4.3 ± 1.5 ] | NA | NA | NA |
| Ghasemi et al., 2019 | NA | Before treatment: [Case group: 29.2 ± 4.58 Control group: 38.40 ± 3.8] After treatment: [Case group: After = 19.68 ± 2.68 / After 2 hr= 19.68 ± 2.68 Control group: After = 28.82 ± 3.02 / After 2 hr= 28.24 ± 2.78] | Before treatment: [Case group: 35.62 ± 9.73 Control group: 24.71 ± 12.13] After treatment: [Case group : After = 79.85 ± 11.66 / After 2 hr= 87.23 ± 11.93  Control group : After = 57.54 ± 25.74 / After 2 hr= 56.96 ± 26.67] | Before treatment: [Case group: mean= 29.93 Control group: mean= 25.75] After treatment: [ Case group: After = 71.24 /After 2 hr= 89.54 Control group: After = 47.71 /After 2 hr= 46.14] | Before treatment: [Case group : 0.77 ± 0.21 Control group : 0.33 ± 0.09] After treatment: [Case group : After= 2.90 ± 0.42 / After 2 hr= 2.90 ± 0.42  Control group : After= 1.77 ± 0.33 / After 2 hr= 1.77 ± 0.33] | NA | Before treatment: [Case group : 14.76 ± 2.29 Control group : 18.20 ± 1.80 (million)] After treatment: [Case group:After = 9.89 /After 2 hr= 9.89 Control group: After = 14.02 /After 2 hr= 14.14] | Before treatment: [Case group: 24.5% Control group: 26.5%] After treatment: [Case group : After= 9.84 ± 1.34 /After 2 hr= 9.84 ± 1.34  Control group : After= 14.41 ± 1.51 / After 2 hr= 14.12 ± 1.39] |
| Canepa et al., 2020 | 150 ng/dL < testosterone < 800 ng/dL | Before treatment:16.6 + 13.1 After treatment: 24.4 + 23.4 | Before treatment : 9.8 + 11.5 (x 106) After treatment: 22.3 + 30.8 (x 106) | Before treatment : 19.5 + 15.6 After treatment: 24.8 + 16.5 | Before treatment : 4.9 + 3.1 After treatment: 7.9 + 4.1 | NA | NA | NA |
| Abdolsamadi et al., 2020 | NA | NA | Case= 37.3 ± 3.3 Control= 12.7 ± 1.7 | Case= 36.6 ± 5.6 Control= 11.11 ± 3.33 | treatment samples : 9.2% control samples : 7.2% | NA | NA | Case= 8.91 ± 4.08 Control= 18 ± 9.4 |
| Santoro et al., 2021 | NA | NA | NA | Before treatment: A= 10.20+ 4.29 After treatment: B= 19.30 + 6.97, C= 19.86+ 7.44 | NA | NA | Before treatment: A= 68.33 ± 7.2 (IN VITRO ), 65.40 ± 3.9 (IN-VIVO) After treatment: B= 75 ± 5.5, C= 70.99 ± 3.3 | NA |
| Azizi et al., 2022 | NA | NA | fresh= 53.5 ± 1.1 Control= 18 ± 3.2 Case= 59.1 ± 1.6 | NA | fresh= 3.07 ± 0.93 Control= 1.84 ± 0.51 Case= 2.87 ± 0.82 | NA | fresh= 59.9 ± 1.6 Control= 55.1 ± 1.1 Case= 62.5 ± 1.6 | fresh= 42.5% Control= 51.75% Case= 25.5% |
| De Leo et al., 2022 | Before treatment: 4.5 ± 1.6 After treatment: 5.2 ± 1.8 | Before treatment: 7.13 ± 4.24 After treatment: 12.24 ± 7.83 | Before treatment: 33.6 ± 5.5 After treatment: 40.2 ± 5.8 | Before treatment: 22.8 ± 5.9 After treatment: 27.5 ± 6.4 | Before treatment: 2.6 ± 1.4 After treatment: 4.2 ± 1.9 | NA | NA | NA |

**Supplementary Table 5-** Pregnancy data of the studies

| **Author, Year** | **Clinical outcome** |
| --- | --- |
| Korosi et al., 2017 | Fertilization index (case) : 84.8 (134/158) Fertilization index (control) : 60.5 (66/109) Number of transferred embryos (case): 1.9 ± 0.4 Number of transferred embryos (control) : 2.0 ± 0.6 Number of pregnancies (case) : 11 Number of pregnancies (control) :0 Good quality embryos at day 3 (case) : 54.7 Good quality embryos at day 3 (control) : 32 |
| Ghasemi et al., 2019 | Infertility duration [ case group :3.46 ± 2.71, control group : 2.85 ± 2.64] pregnancy rate[ case group: 15.01% , control group: 8.16%] |
| Canepa et al., 2020 | within 6 months following Sinopol discontinuation, the partner got pregnant in 40 cases (6 spontaneously, 4 with intra-uterine insemination, and 30 after ICSI or FIVET treatment) |
| De Leo et al., 2022 | Group A : Number retrieved oocytes =9.8 ± 3.5 Number MII oocytes =7.9 ± 2.7 Fertilization rate (%)= 87.3 ± 15.7 Pregnancy rate (%)=19.6 ± 3.7 Group B : Number retrieved oocytes= 9.2 ± 3.3 Number MII oocytes=7.5 ± 2.4 Fertilization rate (%)=74.3 ± 22.6 Pregnancy rate (%)= 17.2 ± 2.9 |

**Supplementary Figure 1.** Risk of bias assessment for each included study

**Supplementary Figure 2-** Funnel plot for total sperm motility


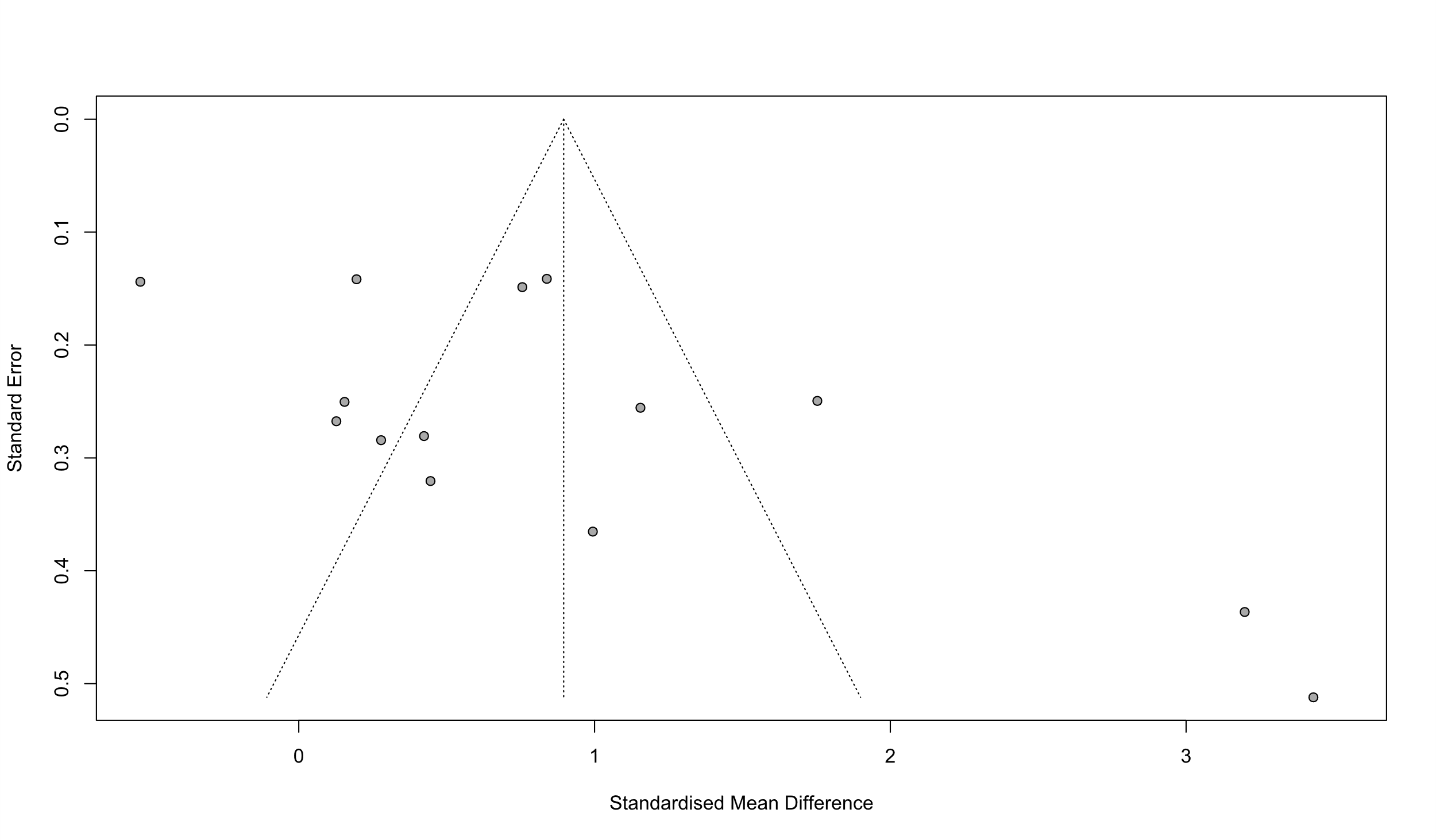


**Supplementary Figure 3-** Funnel plot for progressive sperm motility

1. Ghasemi, A., et al., *The effect of Myo-inositol on sperm parameters and pregnancy rate in oligoasthenospermic men treated with IUI: A randomized clinical trial.* Int J Reprod Biomed, 2019. **17**(10): p. 749-756.


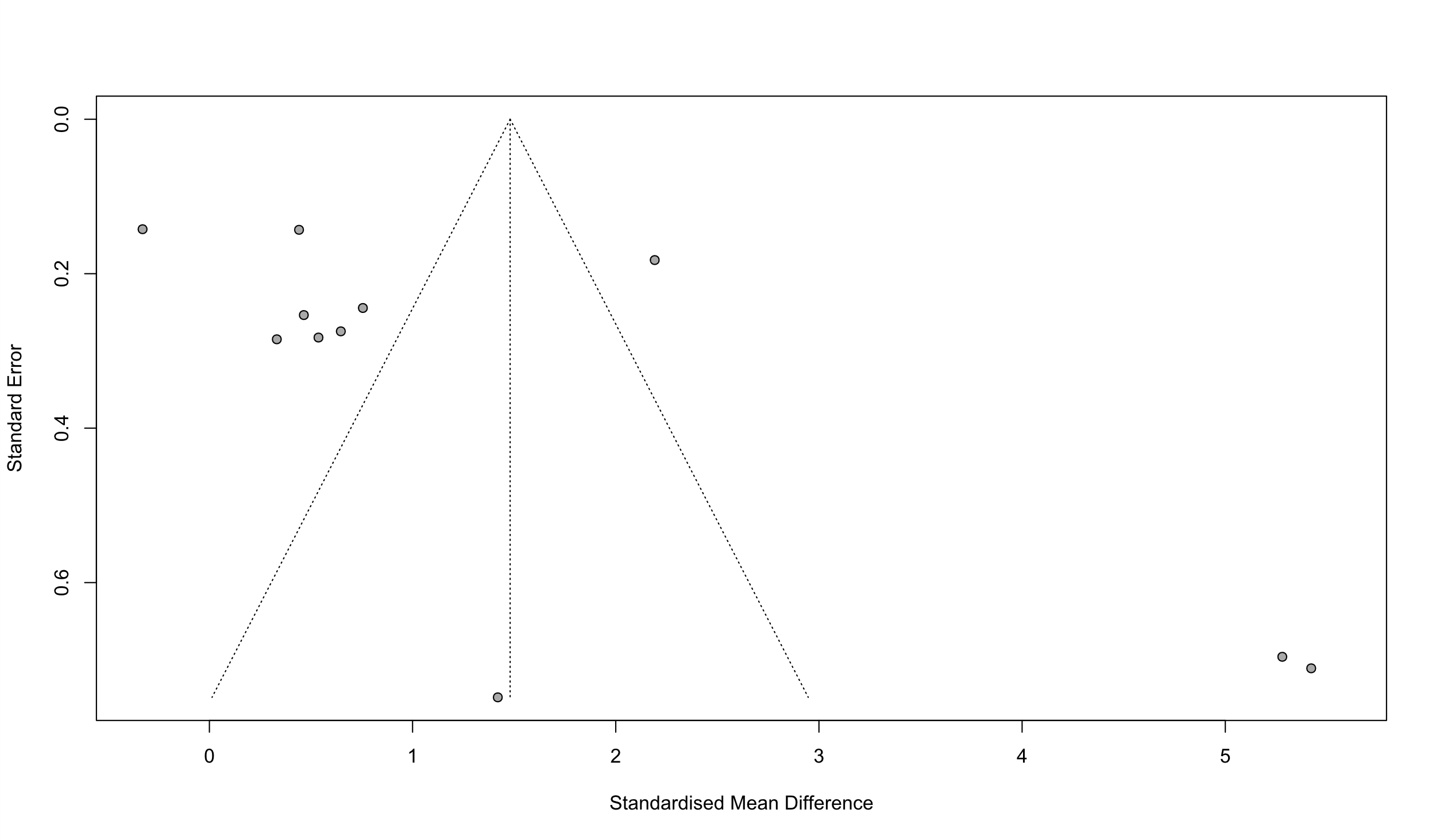

Supplement: Supplementary file 1 — Data S1: [file FSN3-12-8515-s001.docx]
